# Supplementary material for: Identification of a Dexamethasone Mediated Radioprotection Mechanism Reveals New Therapeutic Vulnerabilities in Glioblastoma
Source: Cancers (Basel). 2021 Jan 19;13(2):361. doi: 10.3390/cancers13020361 (PMC7836009; doi:10.3390/cancers13020361)
Supplement: Supplementary file 1 [file cancers-13-00361-s001.zip › cancers-1091321-supplementary/cancers-1091321_supplementary_conversion/SUPPL Figures REVISION corrected-2.pdf]

Supplementary Figure 1

Biodonostia cohort - Patient Information

Basal corticoids (dexamethasone)	RT treatment	Number of patients	Total patient number
Yes	Yes	93	285
	No	51	
No	Yes	114	
	No	27	

Figure S1 - Patient information of the Biodonostia cohort

285 patients (light green) seen at the Donostia University Hospital, San Sebastian and diagnosed with primary glioblastoma grade IV according to the WHO criteria were included in the study. 144 patients had not received DEXA (white), 141 patients received DEXA (red). 207 patients underwent radiotherapy, 78 patients had not undergone radiotherapy.

Supplementary Figure 2

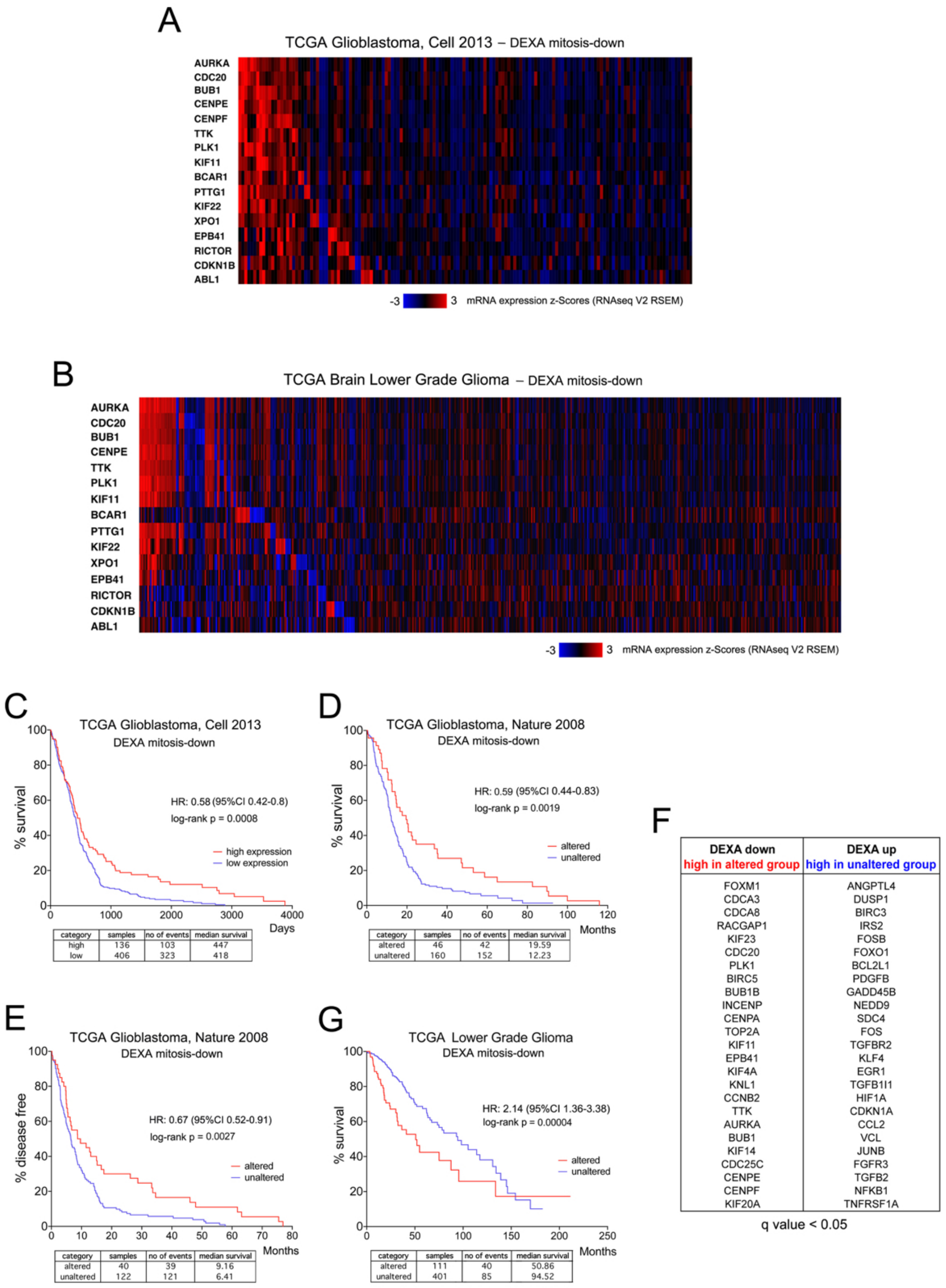

Figure S2 - A DEXA-down signature correlates with poor survival and faster relapse

A Heatmap of mRNA expression of mitosis-control genes down-regulated by DEXA in the TCGA GBM data set (1) generated in cBioportal (2, 3). **B** Heatmap of mRNA expression of mitosis-control genes down-regulated by DEXA in the TCGA lower grade glioma data set (4) generated in cBioportal. **C** Kaplan-Meier analysis of the TCGA GBM patient cohort (1). Differences in overall survival for patients whose tumours express high or low levels of a DEXA-down signature, which includes PLK1, KIF11, XPO1, BCAR1, EPB41, RICTOR, CDKN1B are shown. The signature was analysed in PROGgeneV2 (5) and survival data extracted and analysed in GraphPad Prism. **D** and **E** Kaplan-Meier analysis of the TCGA GBM patient cohort (6). Differences in overall survival **D** and progression free survival **E** for patients whose tumours

Supplementary Figure 2

express high (altered) or low (unaltered) levels of the DEXA-down signature are shown. The signature wasanalysed for mRNA expression (z-score 2.0) in cBioportal and survival data extracted and analysed in GraphPad Prism. **F** List of genes expressed in tumours identified for high (altered) or low (unaltered) levels of the DEXA-down signature. High expression in the altered group of genes, which are down-regulated by DEXA and high expression in the unaltered group of genes, which are up-regulated by DEXA are indicated. This indicates that DEXA is driving an expression profile represented in the unaltered group, which correlates with poorer survival and faster relapse. **G** Kaplan-Meier analysis of the TCGA Brain lower grade glioma patient cohort (4). Differences in overall survival for patients whose tumours express high (altered) or low (unaltered) levels of the DEXA-down signature are shown. The signature was analysed for mRNA expression (z-score 2.0) in cBioportal and survival data extracted and analysed in GraphPad Prism.

References

1. Brennan CW, Verhaak RG, McKenna A, Campos B, Noushmehr H, Salama SR, et al. The somatic genomic landscape of glioblastoma. Cell. 2013;155(2):462-77.
2. Cerami E, Gao J, Dogrusoz U, Gross BE, Sumer SO, Aksoy BA, et al. The cBio cancer genomics portal: an open platform for exploring multidimensional cancer genomics data. Cancer Discov. 2012;2(5):401-4.
3. Gao J, Aksoy BA, Dogrusoz U, Dresdner G, Gross B, Sumer SO, et al. Integrative analysis of complex cancer genomics and clinical profiles using the cBioPortal. Sci Signal. 2013;6(269):pl1.
4. Cancer Genome Atlas Research N, Brat DJ, Verhaak RG, Aldape KD, Yung WK, Salama SR, et al. Comprehensive, Integrative Genomic Analysis of Diffuse Lower-Grade Gliomas. N Engl J Med. 2015;372(26):2481-98.
5. Goswami CP, Nakshatri H. PROGgeneV2: enhancements on the existing database. BMC Cancer. 2014;14:970.
6. Cancer Genome Atlas Research N. Comprehensive genomic characterization defines human glioblastoma genes and core pathways. Nature. 2008;455(7216):1061-8.

Supplementary Figure 3

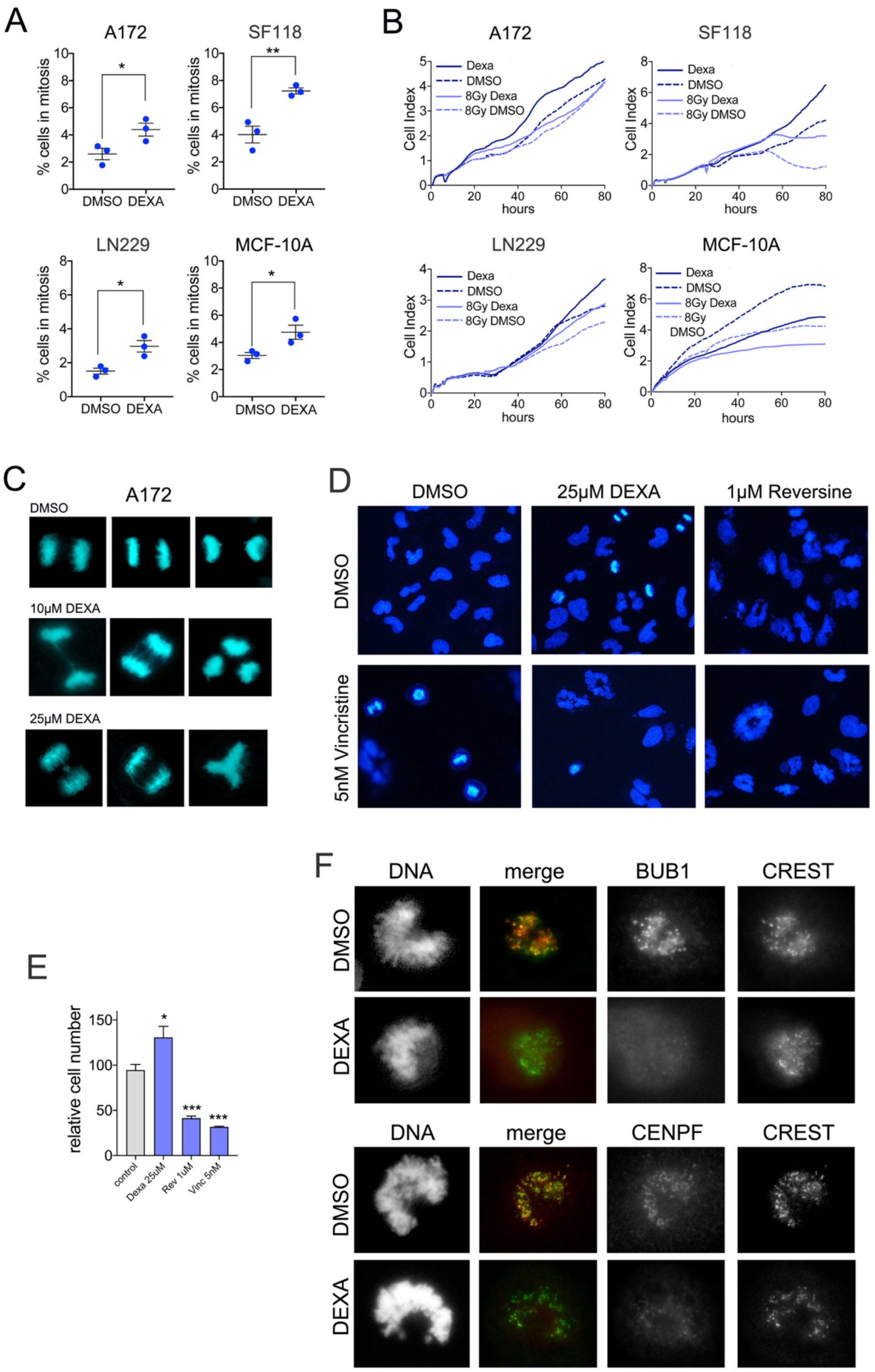

Figure S3 - Dexamethasone induces mitotic errors and overrides the SAC

A % cells in mitosis, quantified (n = 3 experiments) 48h after addition of 25µM DEXA. **B** iCELLigence™ analysis of indicated cell lines either non-radiated or radiated with 8Gy in the absence or presence of 25µM DEXA. **C** A172 cells treated with DMSO or 25µM DEXA for 48h were stained with Hoechst 33258 and imaged. **D** T98G cells were treated with 5nM vincristine either alone or in the presence of 1µM reversine or 25 µM DEXA. After 48h cells were stained with Hoechst 33258 and imaged. **E** In parallel cells were analysed for colony formation (n =3); DMSO treated cells served as control. **F** Immunofluorescence images of T98G cells in prometaphase stained to detect the kinetochore marker CREST (green), and BUB1 (red) or CENPF (red) 36h after addition of 25µM DEXA. DMSO served as control.

Supplementary Figure 4

A

correlation	Pearson	p value
STAT5B vs SRC	0.64	3.24 e -19
STAT5B vs FYN	0.68	7.78 e -22
STAT5B vs YES	0.69	3.29 e -23
SRC vs BCL2L1	0.43	3.48 e -8
FYN vs BCL2L1	0.47	1.15 e -9
YES vs BCL2L1	0.51	3.18 e -11
SRC vs MCL1	0.44	1.97 e -8
FYN vs MCL1	0.52	8.60 e -12
YES vs MCL1	0.70	2.27 e -23
PDGFRA vs STAT5B	0.25	2.94 e -3
PDGFRB vs STAT5B	0.52	5.39 e -11

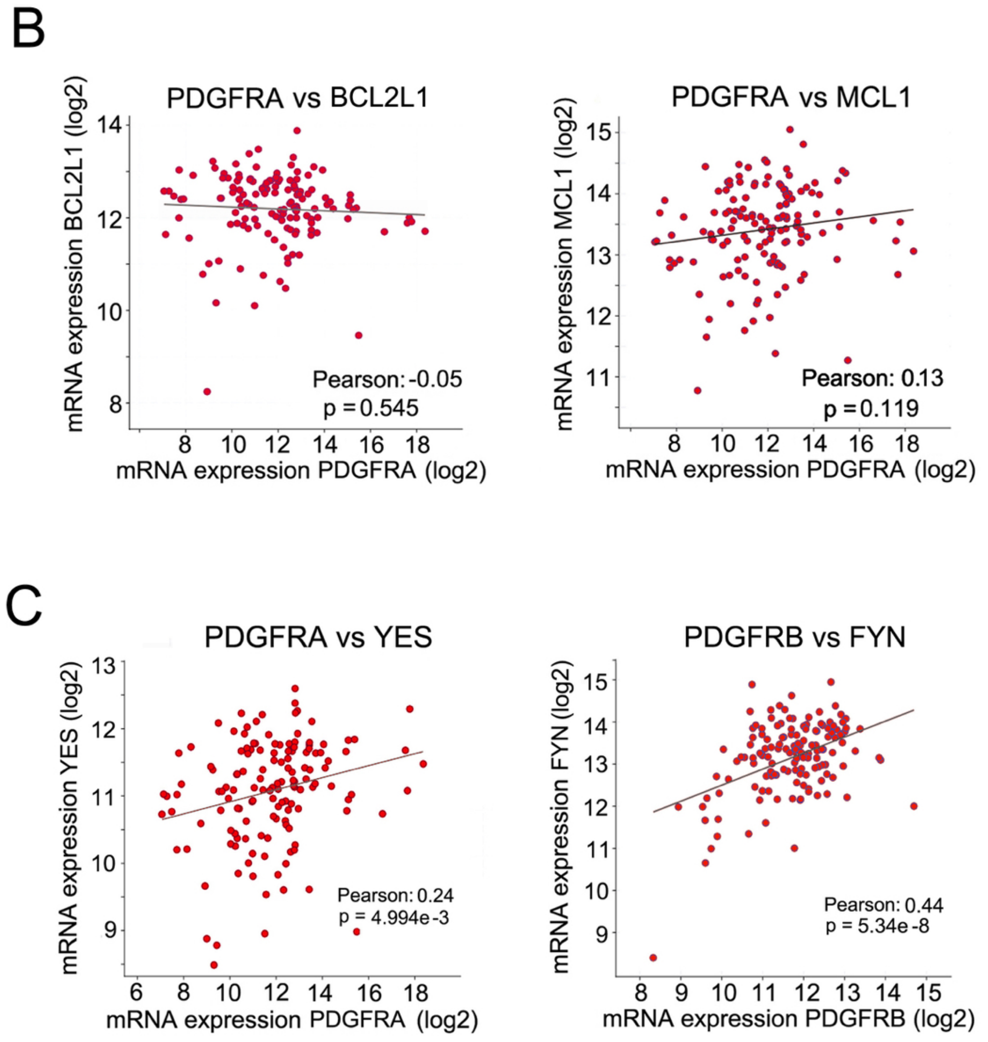

Figure S4 - Co-expression of a PDGFR-STAT5-SFK-BCL2/MCL1 network in the TCGA patient cohort

A Co-expression analysis of the indicated genes performed in the TCGA Glioblastoma dataset (red = SFK; green = BCL2L1; blue = MCL1). **B** Co-expression of PDGFRA with BCL2L1 and MCL1 in the TCGA Glioblastoma dataset. **C** Co-expression of PDGFRA with YES1 and PDGFRB with FYN in the TCGA Glioblastoma dataset.

Supplementary Figure 5

A

	T98G	SF188	A172	LN229	U251
Ponatinib	91 nM	224 nM	167 nM	288 nM	437 nM
Dasatinib	286 nM	320 nM	993 nM	>2 μM	>2 μM
Sunitinib	2.5 μM	1.8 μM	3.4 μM	1.5 μM	3.4 μM
	PDGFRB high		PDGFRB high	PDGFA amp	PDGFRA amp
	EGFR high		EGFR amp	EGFR amp	
		NF1 del		NF1 low	NF1 ins

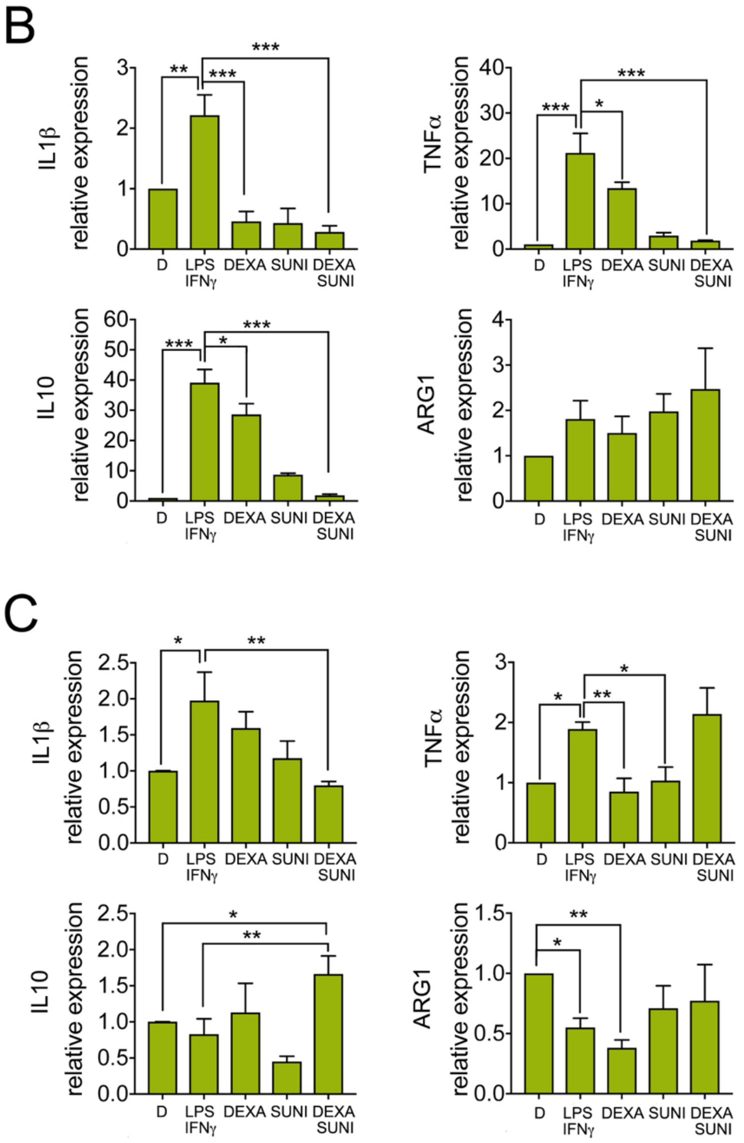

Figure S5 - Sunitinib enhances effects of DEXA on microglial cells

A IC50 values for ponatinib, sunitinib and dasatinib for a panel of GBM cell lines (beige) using colony formation assays. The mutation/expression status of PDGFR (red), EGFR (blue) ad NF1 (grey) is indicated. **B** qRT-PCR analysis for the indicated genes in microglia cells treated with LPS (100ng/ml), IFNγ (50µg/ml) for 6h, followed by addition of DEXA (10µM) or sunitinib (5µM) for 18h as indicated. **C** qRT-PCR analysis for the respective genes in astrocytes treated as described in **B**. Data are represented as mean fold change of at least three repeats relative to DMSO treated control cells.

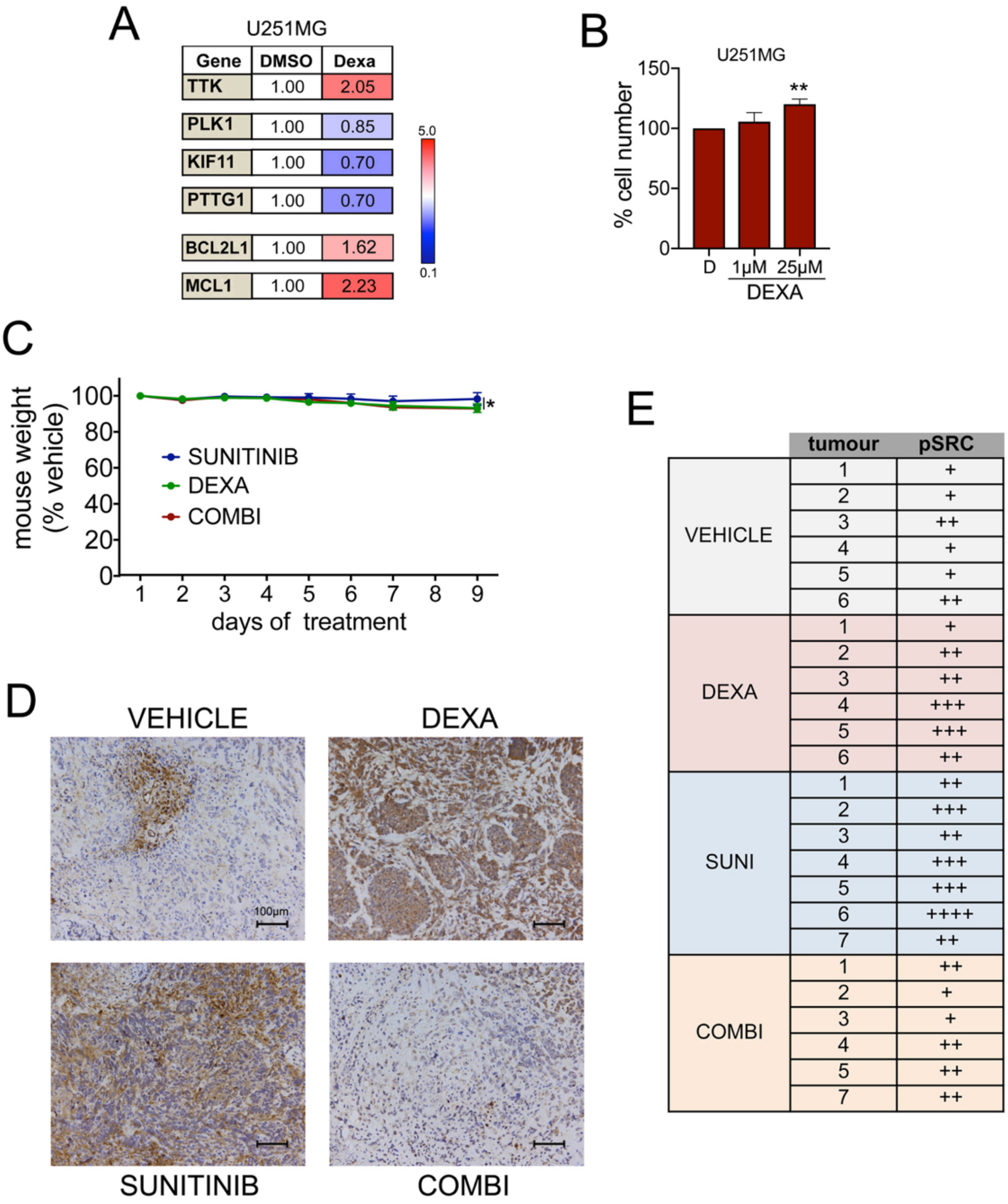

Figure S6 - Sunitinib inhibits the growth promoting effects of DEXA

A qRT-PCR analysis for the indicated genes in U251MG cell lines treated with 25µM DEXA for 18h. Data are represented as mean fold change of triplicates relative to DMSO treated control cells (=1). **B** Quantification of the relative cell number of U251MG cells grown in the absence or presence of 25µM DEXA. Data represent the mean ± SEM (n ≥ 3). **C** Relative weight of mice (n ≥ 6 mice/group) treated as indicated: sunitinib (40mg/kg/qd), DEXA (0.3mg/kg/qd). The weight of vehicle treated mice on day 1 was set 100%. **D** Immunohistochemistry for phosphor-SRC in control tumours (vehicle) and tumours treated with DEXA, sunitinib or the combination of both (COMBI). Scale bar: 100 µm. **E** Quantification of phosho-SRC signal in the indicated tumours. Grey: VEHICLE; pink: DEXA; blue: SUNI; orange: COMBI. + = weak, ++ = medium, +++ = high, ++++ = very high.
