# Supplementary figures and images for: Identification of a Dexamethasone Mediated Radioprotection Mechanism Reveals New Therapeutic Vulnerabilities in Glioblastoma
Source: Cancers (Basel). 2021 Jan 19;13(2):361. doi: 10.3390/cancers13020361 (PMC7836009; doi:10.3390/cancers13020361)

**Supplementary Material – Uncropped Western blots**

Figure 3C

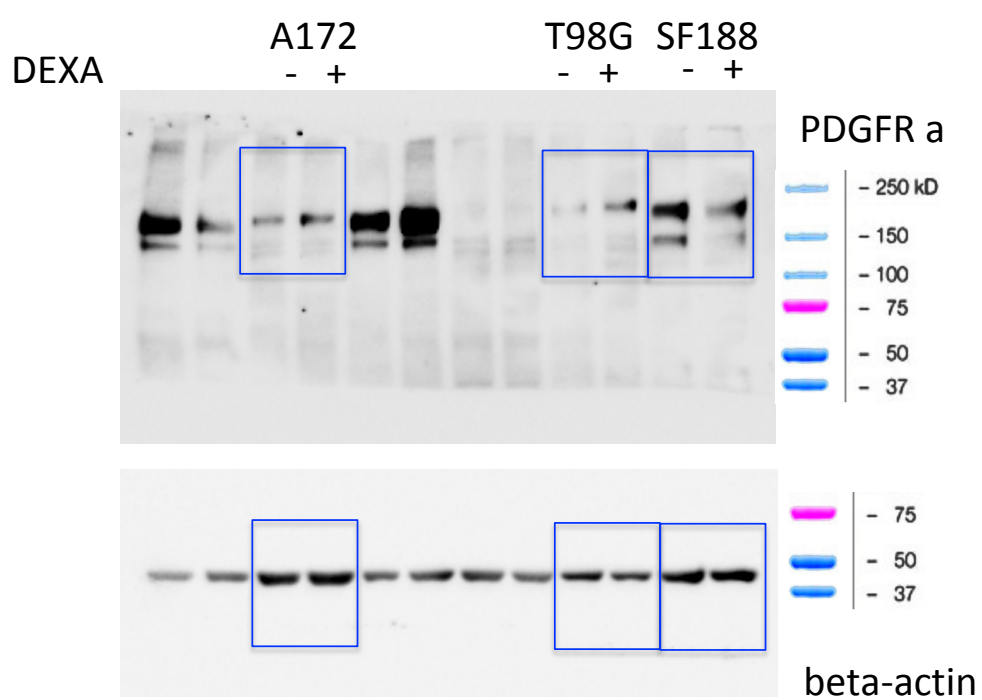

Figure 3C

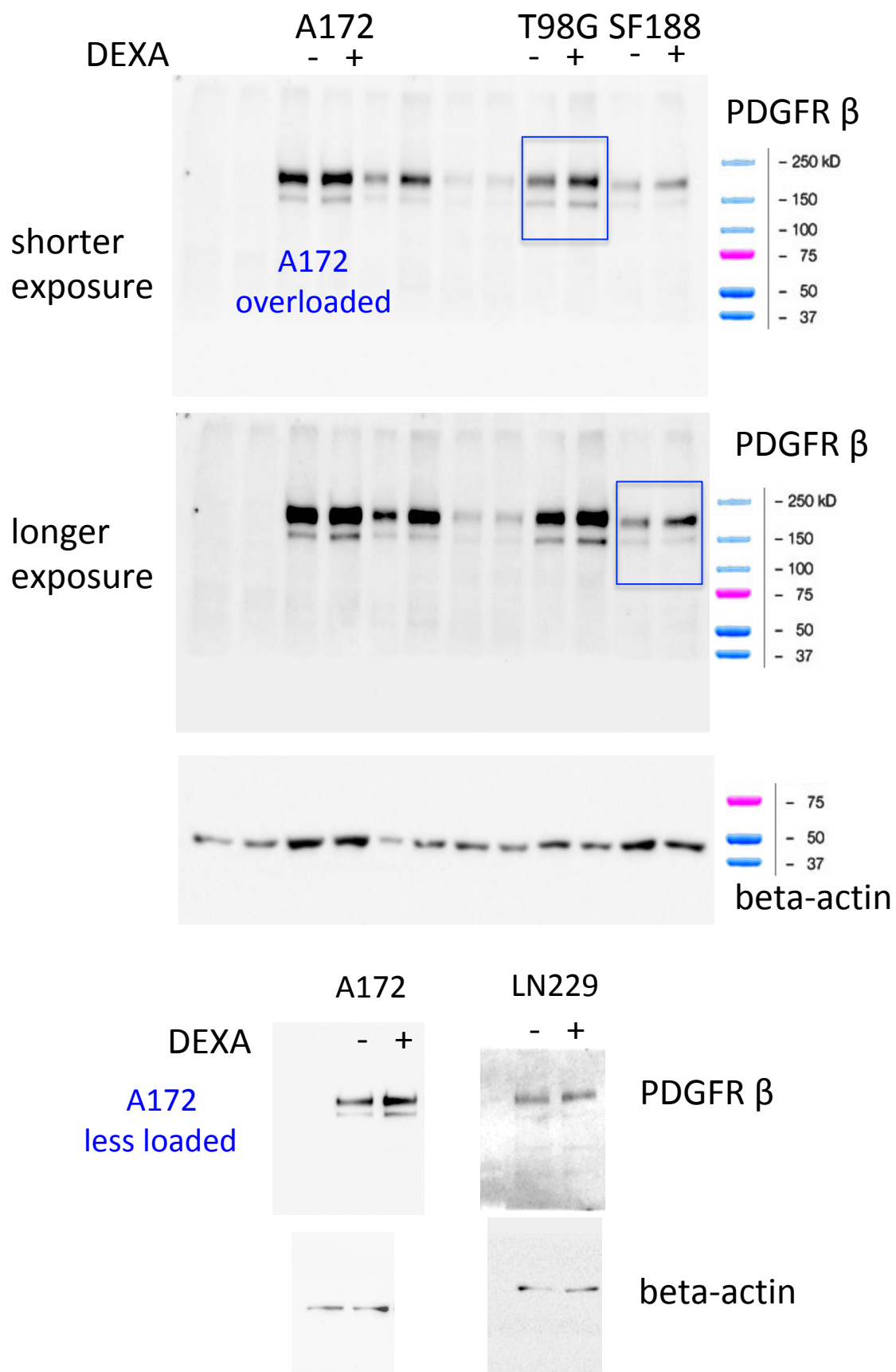

Figure 3D

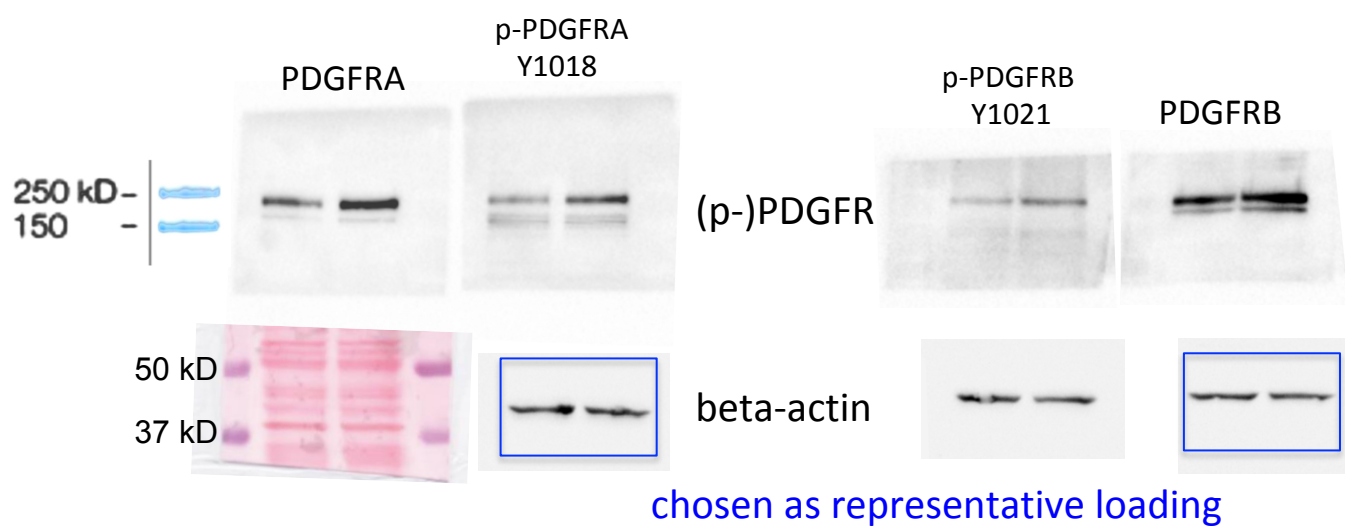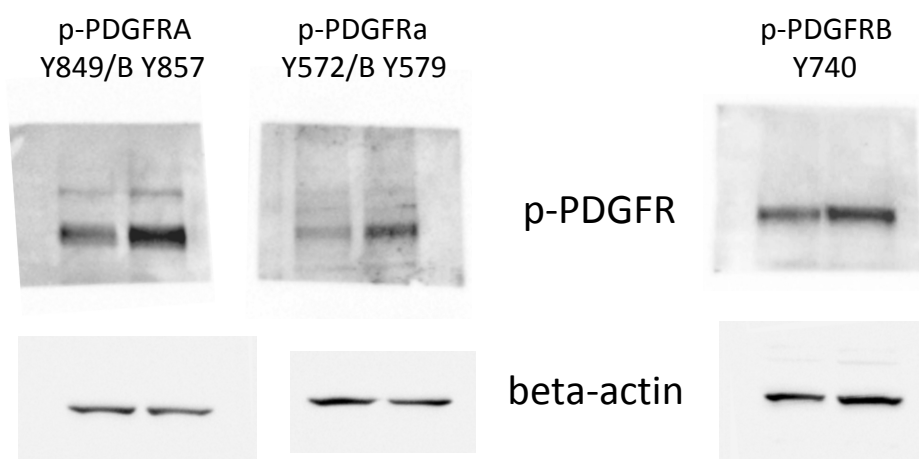

Figure 3E

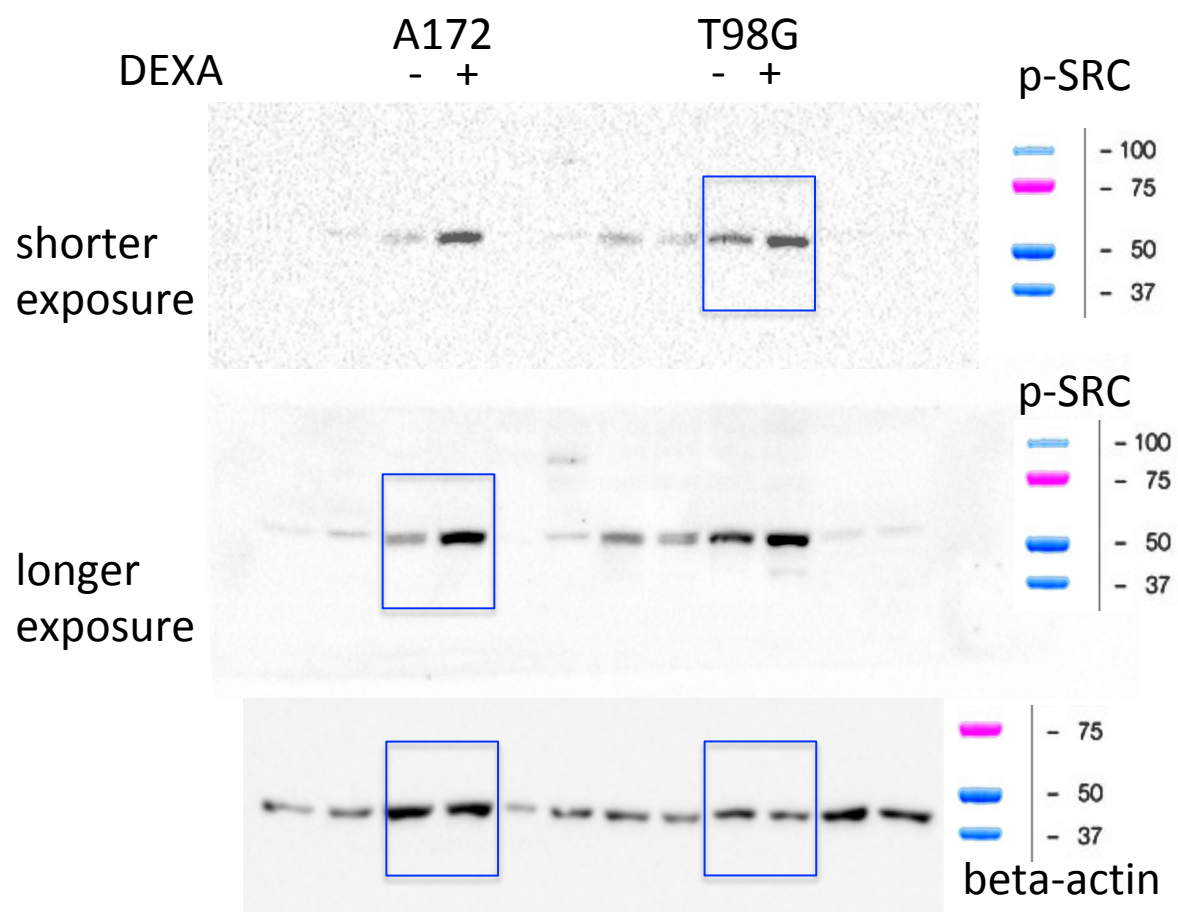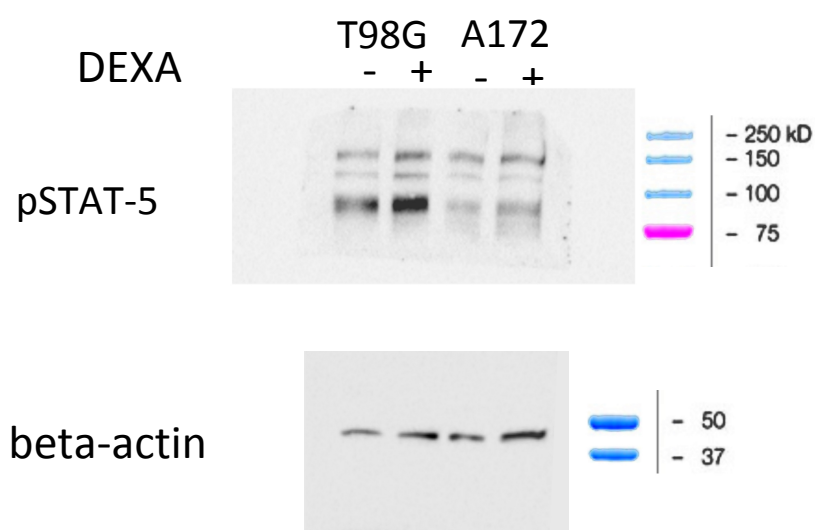

Supplement: Supplementary file 1 [file cancers-13-00361-s001.zip › cancers-1091321-supplementary/cancers-1091321_supplementary_conversion/SUPPL MATERIAL uncropped blots.pdf]
